# Is the Poly (L- Lactide- *Co*– Caprolactone) Nanofibrous Membrane Suitable for Urinary Bladder Regeneration?

**DOI:** 10.1371/journal.pone.0105295

**Published:** 2014-08-27

**Authors:** Marta Pokrywczynska, Arkadiusz Jundzill, Jan Adamowicz, Tomasz Kowalczyk, Karolina Warda, Marta Rasmus, Lukasz Buchholz, Sandra Krzyzanowska, Pawel Nakielski, Tomasz Chmielewski, Magdalena Bodnar, Andrzej Marszalek, Robert Debski, Malgorzata Frontczak-Baniewicz, Grzegorz Mikułowski, Maciej Nowacki, Tomasz A. Kowalewski, Tomasz Drewa

**Affiliations:** 1 Chair of Regenerative Medicine, Department of Tissue Engineering, Nicolaus Copernicus University in Torun, Ludwik Rydygier Medical College in Bydgoszcz, Bydgoszcz, Poland; 2 Department of Theory of Continuous Media, Institute of Fundamental Technological Research, Polish Academy of Sciences, Warsaw, Poland; 3 Department of Mechanics and Physics of Fluids, Institute of Fundamental Technological Research, Polish Academy of Sciences, Warsaw, Poland; 4 Department of Clinical Pathomorphology, Nicolaus Copernicus University in Torun, Ludwik Rydygier Medical College in Bydgoszcz, Bydgoszcz, Poland; 5 Department of Tumor Pathology, Center of Oncology, Poznan University of Medical Sciences, Poznan, Poland; 6 Department of Pediatrics, Hematology and Oncology, Nicolaus Copernicus University in Torun, Ludwik Rydygier Medical College in Bydgoszcz, Bydgoszcz, Poland; 7 Electron Microscopy Platform, Mossakowski Medical Research Centre, Polish Academy of Sciences, Warsaw, Poland; 8 Department of Intelligent Technologies, Institute of Fundamental Technological Research, Polish Academy of Sciences, Warsaw, Poland; 9 Department of Urology, Nicolaus Copernicus Hospital, Torun, Poland; Texas A&M University Baylor College of Dentistry, United States of America

## Abstract

The purpose of this study was to compare: a new five-layered poly (L–lactide–*co*–caprolactone) (PLC) membrane and small intestinal submucosa (SIS) as a control in rat urinary bladder wall regeneration. The five-layered poly (L–lactide–*co*–caprolactone) membrane was prepared by an electrospinning process. Adipose tissue was harvested from five 8-week old male Wistar rats. Adipose derived stem cells (ADSCs) were seeded in a density of 3×10^6^ cells/cm^2^ onto PLC membrane and SIS scaffolds, and cultured for 5-7 days in the stem cell culture medium. Twenty male Wistar rats were randomly divided into five equal groups. Augmentation cystoplasty was performed in a previously created dome defect. Groups: (I) PLC+ 3×10^6^ADSCs; (II) SIS+ 3×10^6^ADSCs; (III) PLC; (IV) SIS; (V) control. Cystography was performed after three months. The reconstructed urinary bladders were evaluated in H&E and Masson's trichrome staining. Regeneration of all components of the normal urinary bladder wall was observed in bladders augmented with cell-seeded SIS matrices. The urinary bladders augmented with SIS matrices without cells showed fibrosis and graft contraction. Bladder augmentation with the PLC membrane led to numerous undesirable events including: bladder wall perforation, fistula or diverticula formation, and incorporation of the reconstructed wall into the bladder lumen. The new five-layered poly (L–lactide–*co*–caprolactone) membrane possesses poorer potential for regenerating the urinary bladder wall compared with SIS scaffold.

## Introduction

The small intestine is commonly used for urinary tract reconstruction, either for bladder augmentation, replacement, or urinary diversion. Unfortunately, these techniques are associated with numerous complications [1]. Many natural and synthetic biomaterials such as plastic mold, gelatin sponge, Japanese paper, preserved dog bladder, lyophilized human dura, bovine pericardium, small intestinal submucosa, bladder acellular matrix, or composites of collagen and polyglycolic acid were used for human urinary bladder regeneration with a wide range of outcomes [2]. However, the ideal biomaterial for urinary bladder reconstruction has not been found thus far. The scaffolds used in tissue engineering should mimic the ability of the extracellular matrix (ECM) to regulate cell functions such as cell division, differentiation, and apoptosis [3]. The biomaterial intended for urinary bladder reconstruction should be not only biocompatible and biodegradable but also waterproof, flexible, elastic and able to provide good mechanical strength [4].

Decellularized extracellular matrices such as small intestinal submucosa (SIS) or bladder acellular matrix (BAM) have been widely used for urinary bladder reconstruction in various models [5]–[8]. However, acellular matrices have some disadvantages including immunogenicity, lot- to- lot variability, and inadequate biomechanical properties [9]. Consequently, there is a growing interest in developing new synthetic biomaterials for urinary bladder regeneration [10], [11]. Aliphatic polyesters such as poly(glycolide) (PGA), poly(lactide) (PLA), poly(ε-caprolactone) (PCL), and their copolymers have been widely used in tissue engineering with favorable results [12]–[16]. Several *in vitro* studies revealed the significant potential of PLC membranes for tissue engineering in urology [17], [18]. This data has encouraged us to produce the PLC membrane for urinary bladder wall regeneration. The electrospinning process is a promising way for providing nanofibrous scaffolds, which closely mimic the natural extracellular matrix structure [19]. Matrices made of electrospun nanofibers when compared to natural collagen matrices have certain advantages: they are non-allergenic, do not involve risk of transferring diseases (e.g. prions), and are easy to prepare, handle, and store. Urine is extremely cytotoxic for stem cells [20], [21]. Therefore, the separation of stem cells from the toxic environment of urine seems to be crucial for urinary bladder tissue engineering. In this experiment, we evaluate poly (L–lactide–*co*–caprolactone), a new nanofibrous and durable membrane, designed to isolate cells from urine in tissue engineered urinary bladders.

## Materials and Methods

### Synthesis of five-layered poly (L-lactide-co-caprolactone) membrane via an electrospinning technique

**Figure 1:**
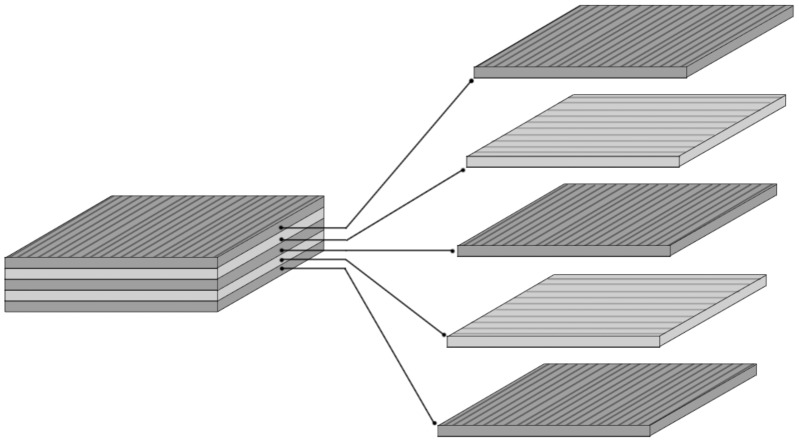
Scheme of five-layered electrospun membrane structure.

Poly (L–lactide–*co*–caprolactone) (PLC) (Purac, Netherlands), chloroform (CHCl_3_) (POCh, Poland), N,N-dimethylformamide (DMF)(POCh, Poland), sodium bicarbonate (NaHCO_3_) (POCh, Poland) were used as supplied, without further purification. Nanomaterials were prepared using an electrospinning process described by Li et al [22]. Briefly, PLC was dissolved in a mixture of CHCl_3_ and DMF (mass proportion 16∶1) to form 9% solution and left overnight. Electrospinning was conducted in a custom-made polycarbonate chamber of approximate volume of 1 m^3^. The electrospinning setup consisted of custom-made voltage power supply (HVPS) with adjustable output voltage. The electrospun solution was transferred from a metering syringe pump (New Era Pumping Systems, Great Britain) through a hydraulic system to a nozzle made of blunt needle (outer volume 0.4 mm) connected to HVPS output. Each element of the setup has a commercial market counterpart. Electrically grounded aluminum foil attached to a fast rotating polypropylene mandrel served as a target. After collection of each consecutive layer of directed nanofibrous material, the foil was detached from the mandrel, positioned orthogonally to a previous direction, and attached back to the mandrel. Material of a plywood-like structure was formed after 5 repetitions of the fiber collection process (Fig. 1). Electrospinning parameters were as follows: voltage- 20 kV, polymer solution flow- 800 µl/h, spinneret to collector distance- 20 cm, mandrel speed- 4100 rpm, mandrel diameter- 60 mm.

### Modification of five-layered poly (L-lactide-co-caprolactone) membrane

The surface of nanomaterial was modified by treatment with a 0.5 M solution of sodium bicarbonate dissolved in deionized water. Filter paper was soaked with a treatment solution and placed on top of the membrane for 30 min. The results were measured using a goniometric method by measuring contact angle of 2 µl droplet of water placed on a membrane before and after modification (OCA 20, DataPhysics Instruments GmbH, Filderstad, Germany). ImageJ software (NIST, USA) was used to evaluate data.

### Poly (L-lactide-co-caprolactone) membrane nanofiber thickness and pore size

The ImageJ software was used to assess fiber diameters. For each sample of nanofiber material, three SEM images were acquired and 33 measurements were taken from each image. The measurement was made perpendicular to the fiber's axis. Scaffold porosity (ε) was calculated by the method given by Hu [23] using density of the scaffold (ρ_scafflold_) and density of neat PLC (ρ_PLC_): ε = 1- (ρ_scafflold_/ρ_PLC_). Characteristic 3-dimensional pore diameter (d_3D_) was calculated by the method given by Tomadakis and Robertson [24] on the basis of scaffold porosity (ε) and mean fiber diameter (ω): d_3D_ = − ω/ln(ε)

### Analysis of the mechanical properties of five-layered poly (L-lactide-co-caprolactone) membrane

The tensile tests were conducted on a load frame of a servohydraulic material testing machine (MTS 242.01 actuator, Eden Prairie, USA). The specimen (25 mm length, 12.5 mm width) was mounted into flat grips with gauge base 12.5 mm. During the test, the specimen was longitudinally extracted at a rate of 0.3 mm/s to failure. The grip travel and specimen load were continuously measured over the test procedure duration with a precision force transducer (Interface, model 1500, measuring range 125 N, resolution 0.0625 N) and MTS system linear variable differential transformer (measuring range 100 mm, resolution 0.01 mm). Stress/strain curve for the specimen was generated and the ultimate tensile strength, maximum strain and elastic modulus were determined. The strength (MPa) was calculated by dividing the value of failure load by initial cross-sectional area of the specimen. The maximum strain (the strain value corresponding to the ultimate stress) was calculated as the elongation of the specimen divided by the initial gauge length (mm/mm). The elastic modulus (MPa) was defined as the slope of the linear regions of the stress/strain curve.

### Ethics Statement

This study was carried out in strict accordance with the recommendations in the Guide for the Care and Use of Laboratory Animals of the National Institutes of Health [25]. The protocol was approved by the Nicolaus Copernicus University Ethics Committee (no. 4/2012).

### Adipose Derived Stem Cell Isolation and Culture

Adipose tissue was harvested from the retroperitoneal space from five syngeneic 8-week male Wistar rats. The animals were euthanized with an overdose of ketamine 75 mg/kg (Biowet, Poland). ADSCs were isolated according to the method described by Safford et al [26]. Briefly, adipose tissue (1 g) was digested in collagenase type I solution (1 mg/ml, Sigma- Aldrich, Germany) for 30 min at 37°C with shaking. The reaction was stopped by adding Dulbecco's Modified Eagle's Medium (DMEM; PAA, Austria), supplemented with 10% fetal bovine serum (FBS; PAA, Austria) and antibiotics (PAA, Austria). The cell suspension was filtered by a 100 µm cell strainer (Becton Dickinson, USA) and centrifuged at 350×g for 5 min. Viable cells were counted via trypan blue staining and seeded on 25 cm^2^ flask in a density of 15×10^3^ cells/cm^2^. ADSCs were cultured in a medium consisting of DMEM supplemented with 10% FBS, fibroblast growth factor (FGF, 10 ng/ml; Sigma- Aldrich, Germany), penicillin/streptomycin (100 U/100 µg/ml) and amphotericin B (100 µg/ml) (PAA, Austria) at 37°C, 5%CO_2_ and 95% humidity until 3^rd^ passage.

### Adipose Derived Stem Cells Characterization

To confirm the ADSCs phenotype, cells were subjected to antigen analysis by flow cytometry. Detached cells from the 3^rd^ passage were washed and re-suspended with phosphate buffered saline (PBS). Approximately 1×10^6^ cells were incubated with monoclonal primary antibodies conjugated with PE or FITC against CD34 (Santa Cruz Biotechnology, Inc, USA; 20 µl/sample), CD44 (Millipore, USA; 10 µl/sample), CD45 (BD, Pharmingen, USA; 0.06 µg/sample) and CD90 (Millipore, USA; 10 µl/sample) for 30 min. Expression level of each surface marker was quantified using an EPICS XL flow cytometer (Beckman Coulter, USA).

The differentiation of ADSCs into adipogenic, osteogenic and chondrogenic lineages was induced by culture in appropriate differentiation media, according to manufacturer's instruction (Invitrogen, USA). Negative control cells were maintained in DMEM/Ham's F-12 supplemented with 10% FBS and antibiotics. Adipogenesis was measured by the accumulation of neutral lipids in fat vacuoles, stained with Oil-Red-O (Sigma- Aldrich, Germany). Osteogenesis was confirmed using Alizarin Red staining (Millipore, USA). Chondrogenic differentiation was evaluated by anti-collagen type II immunocytochemical staining (anti-collagen II clone 6B3, Millipore, USA, 1∶100, 16 h, 4°C).

### Biomaterial Cytotoxicity Assay

Biomaterial cytotoxicity was determined using extract toxicity assay according to ISO-10993 norm. A piece of biomaterial (∼6 cm^2^) was extracted in DMEM supplemented with 10% FBS and antibiotics (1 ml) at 37°C for 120 h. The extract was filtered and stored at 4°C for up to one week. Extract cytotoxicity was determined using the xCELLigence system (Real-Time Cell Analyzer Dual Plate, RTCA DP, Roche Applied Science, Germany). The xCELLigence system is a unique, cellular impedance- based systems that allows the real- time monitoring of cell growth. When adherent cells attach and spread on the sensor surface of an electrode, increases in impedance are recorded. The changes in impedance are expressed as cell index (CI). For this purpose, ADSCs were seeded on E-Plates (4×10^3^ cells/well) and cultured until reaching a log- phase growth in DMEM supplemented with 10% FBS and antibiotics. Then the medium was changed for a fresh one (negative control) or medium supplemented with 25%, 50% and 75% biomaterial extract (PLC25, PLC50, PLC75, SIS25, SIS50, SIS75 respectively). The experiment was continued until ADSCs achieved the plateau growth phase.

### Analysis of Adipose Derived Stem Cells Growth on the poly (L-lactide-co-caprolactone) membrane

ADSCs were seeded on the 1 cm^2^ of poly (L-lactide-*co*-caprolactone) electrospun membrane or on small intestinal submucosa (SIS) (Surgisis, Biodesign, USA) mounted on cell crowns in a density of 3×10^6^ cells/cm^2^, and cultured for 7 or 14 days. ADSCs growth on PLC and SIS scaffolds was assessed by scanning electron microscopy. For this purpose, the specimens were fixed in 2% paraformaldehyde and 2,5% glutaraldehyde in phosphate buffer for 2 h, post-fixed in 1% OsO4 and dehydrated with grades series of ethyl alcohol followed by acetone. Next the specimens were critically-dried and coated with gold particles before observation in scanning electron microscope (JEOL JSM-6390LV, Japan).

### Graft preparation

To prepare the grafts for bladder augmentation, the ADSCs were seeded on PLC or SIS scaffolds and cultured for 5-7 days.

### Augmentation cystoplasty

Twenty syngeneic male Wistar rats weighing between 250 and 300 g were randomly divided into five equal groups. Sixteen rats, anesthetized with sodium pentobarbital (15 mg/kg, i.p., Biowet, Poland) and lidocaine (20 mg/kg, i.m., Polfa, Poland), underwent hemicystectomy and bladder augmentation with approximately 1 cm^2^ of graft. The anastomosis line was marked by 8.0 monofilament non-absorbable marker sutures to identify the graft borders. In the first group, bladders were reconstructed using PLC membrane seeded with 3×10^6^ ADSCs. In the second group, bladders were augmented using SIS seeded with 3×10^6^ ADSCs. In the third and fourth groups, bladders were reconstructed with unseeded PLC and SIS membranes, respectively. In order to achieve a good analgesic effect, the lidocaine was additionally injected (20 mg/kg, i.m.) after the procedure. The fifth group (control) was left intact. Cystography, morphological and histological studies were performed after three months follow-up.

### Cystography

Under general anesthesia with sodium pentobarbital (15 mg/kg, i.p.) and lidocaine (20 mg/kg, i.m.), the animal was placed in supine position with knees flexed. A small, flexible catheter (3 Fr, Galmed, Poland) was gently inserted into the rat's urethra and the urinary bladder was injected with radiocontrast (0.5 ml of 20% iopromide, Bayer Pharma AG, Germany). The cystograms were performed by fluoroscopy (Actube Dental 5D2, exposition 60 kV and 6mAm). The animals were euthanized with an overdose of ketamine (75 mg/kg).

### Histological staining

The bladder samples were fixed in 10% buffered formaldehyde, using routine procedure of tissue processing, and embedded in paraffin. Cross-sections of whole bladders were made. The 4 µm thick paraffin sections were stained with hematoxylin and eosin. The connective tissue components and muscle layer were stained according to Masson staining. The smooth muscle abundance was assessed using the ImageJ program according to the method described previously [5]. The analysis was repeated for nine areas from each of the specimens.

### Statistical analysis

Statistical differences between groups were determined by one-way ANOVA followed by Tamhane' *post hoc* multiple-comparison test (IBM SPSS Statistics, Predictive Solutions, Poland). Statistically significant differences were defined as having *p*<0.05.

## Results

### Synthesis and characterization of five-layered poly (L-lactide-co-caprolactone) membrane

**Figure 2:**
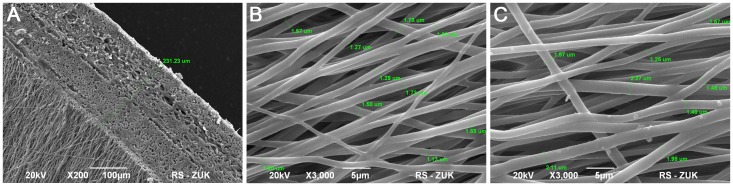
Ultrastructural analysis of the electrospun poly (lactydo-*co*-caprolactone) (PLC) membrane: thickness of five- layered PLC membrane (A), the surface of unmodified PLC membrane (B) the surface of PLC membrane modified with sodium bicarbonate (C). Scanning Electron Microscopy, bar 5 µm, 100 µm.

**Figure 3:**
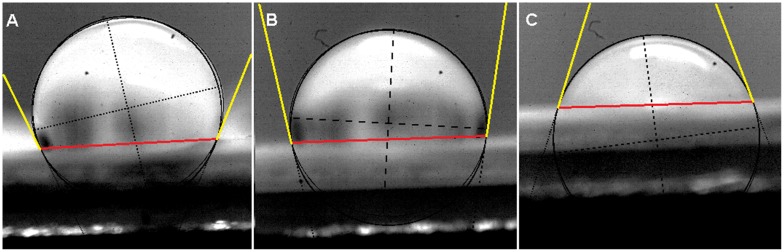
Contact angle measurements: untreated poly (lactydo-*co*-caprolactone) (PLC) membrane, 110° (A), NaHCO_3_ treated PLC membrane: unmodified side- 97° (C) and modified side- 72° (B). Contact angle is the angle between the baseline of the drop (marked in red) and the tangent at the drop boundary (marked in yellow).

The electrospun *five-layered* poly (L-lactide-*co*-caprolactone) membrane thickness was approximately 230 µm (Fig. 2A). The membrane had a micro-porous structure (Fig. 2B). The mean 3D pore diameter was 2.69±0.62 µm. The fiber diameter ranged between 740 nm and 2.27 µm (mean 1.39±0.32 µm). The NaHCO_3_ treatment did not significantly change the thickness of PLC fibers (mean 1.51±0.36 µm) and 3D pore diameter (mean 2,91±0. µm)(p>0.05) (Fig. 2C). The contact angle of PLC membrane before modification was 110 degrees. Sodium bicarbonate (NaHCO_3_) treatment led to a decrease of the contact angle of the PLC membrane by 25°C (Fig. 3). The mechanical properties of the PLC membrane are given in Table 1.

**Table 1:** Tensile properties of the five-layered poly (lactydo-*co*-caprolactone) (PLC) membrane and bladder specimens.

Material	Ultimate tensile strain (mm/mm)	Ultimate tensile strength (MPa)	Elastic modulus R1 (MPa)	Elastic modulus R2 (MPa)
PLC	4.35	11.4	16.0	2.25
Rat bladder (27)	2.03	0.72	-	0.76
Pig bladder (27)	1.66	0.32	-	0.26
Human bladder (27)	0.69	0.27	-	0.25

The elastic range 1 (R1) was demonstrated up to 24% of strain and the elastic range 2 (R2) between 70 % and 429 %.

### Analysis of Phenotype and Multipotent Character of Adipose Derived Stem Cells

**Figure 4:**
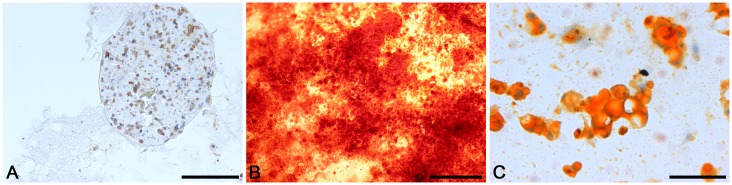
Differentiation potential of adipose derived stem cells: a positive anti-collagen II staining after chondrogenic induction, bar 200 µm (A), Alizarin Red staining after osteogenic induction, bar 500 µm (B), Oil Red O staining after adipogenic induction, bar 20 µm (C).

Flow cytometry confirmed the ADSCs phenotype. ADSCs derived from the third passage were positive for the CD44^+^ (50% of cells) and CD90^+^ (87% of cells) markers and negative for typical endothelial and hematopoietic markers CD34^+^ (16% of cells) and CD45^+^ (13% of cells). ADSCs were able to differentiate into adipocytes, osteoblasts and chondrocytes after cultivation in respective media (Fig. 4). Controls showed negative results.

### Analysis of Biomaterial Cytotoxicity in vitro

**Figure 5:**
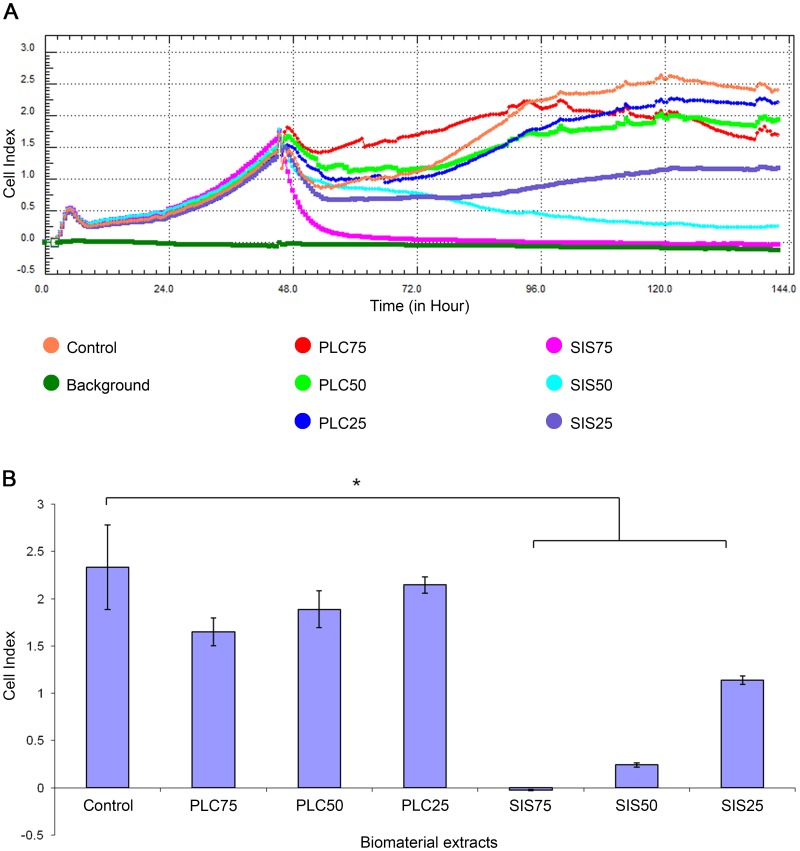
Analysis of poly (lactydo-*co*-caprolactone) (PLC) and small intestinal submucosa (SIS) cytotoxicity using Real Time Cell Analyzer (RTCA). Adipose derived stem cells were treated with 75%, 50% and 25% extracts of PLC (PLC75, PLC50, PLC25 respectively) and SIS (SIS75, SIS50, SIS25 respectively). The results are presented as: cell growth curves (A), mean cell index ± standard deviation after 96 hours of cell incubation with extracts. The statistical significance is shown as * p<0.05 (B)

Real- time cell analysis of the cytotoxicity of the PLC and SIS extracts is presented in Fig. 5A. There was no significant decrease in cell indexes following 96 hours of incubation of ADSCs with PLC25, PLC50 and PLC75 extracts compared to the negative control (p>0.05). However, there was significant reduction in cell indexes following incubation of ADSCs with SIS25, SIS50 and SIS75 extracts compared to negative control (p<0.05) (Fig. 5B).

### Analysis of Growth of Adipose Derived Stem Cells on Five- layered Poly (L-lactide-co-caprolactone) Membrane and Small Intestinal Submucosa

**Figure 6:**
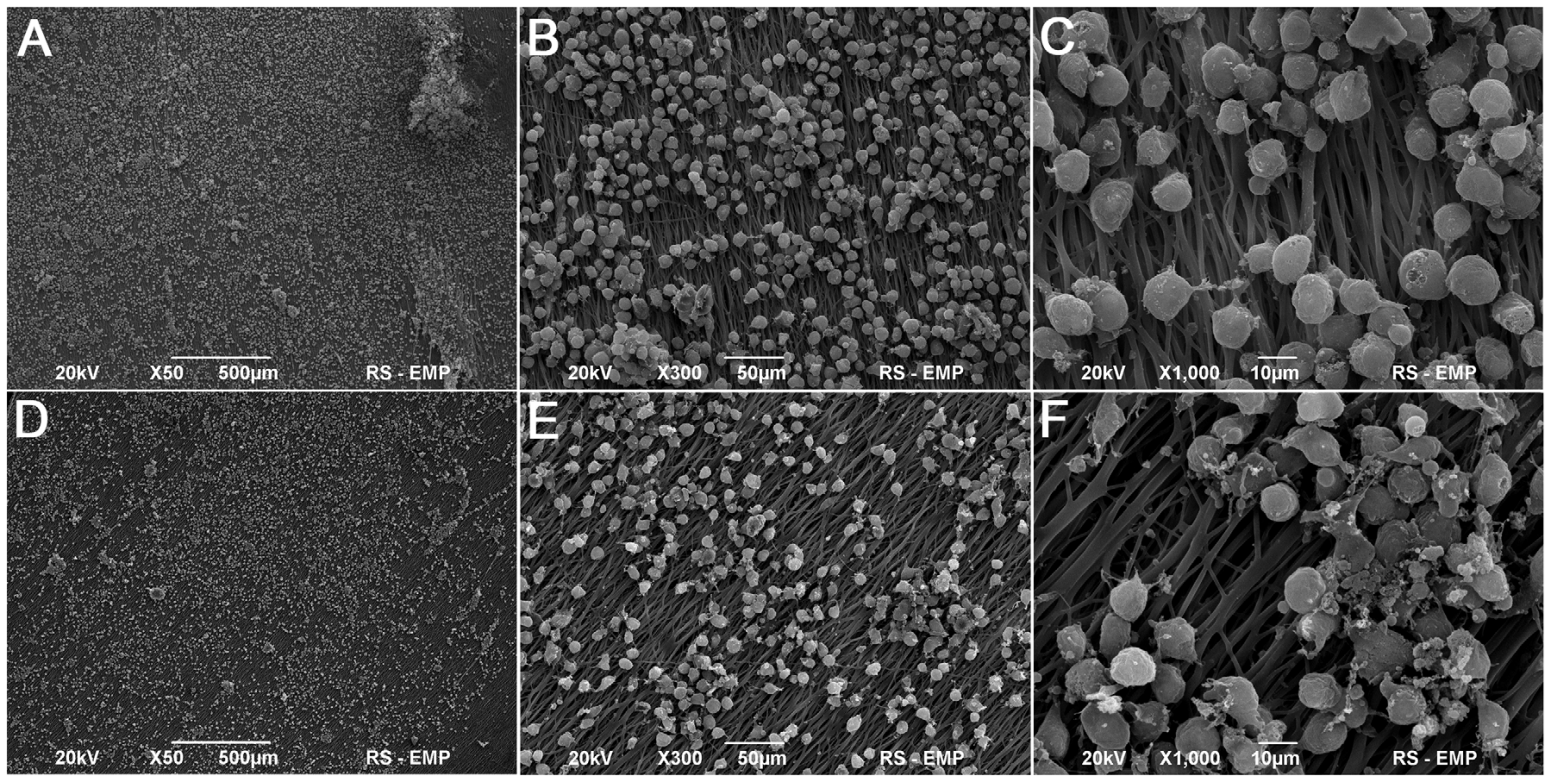
Poly (lactydo-*co*-caprolactone) membrane seeded with adipose derived stem cells (ADSCs), 7^th^ (A,B,C) and 14^th^ day of culture (D,E,F), Scanning Electron Microscopy, bar 500 µm, 50 µm, 10 µm.

**Figure 7:**
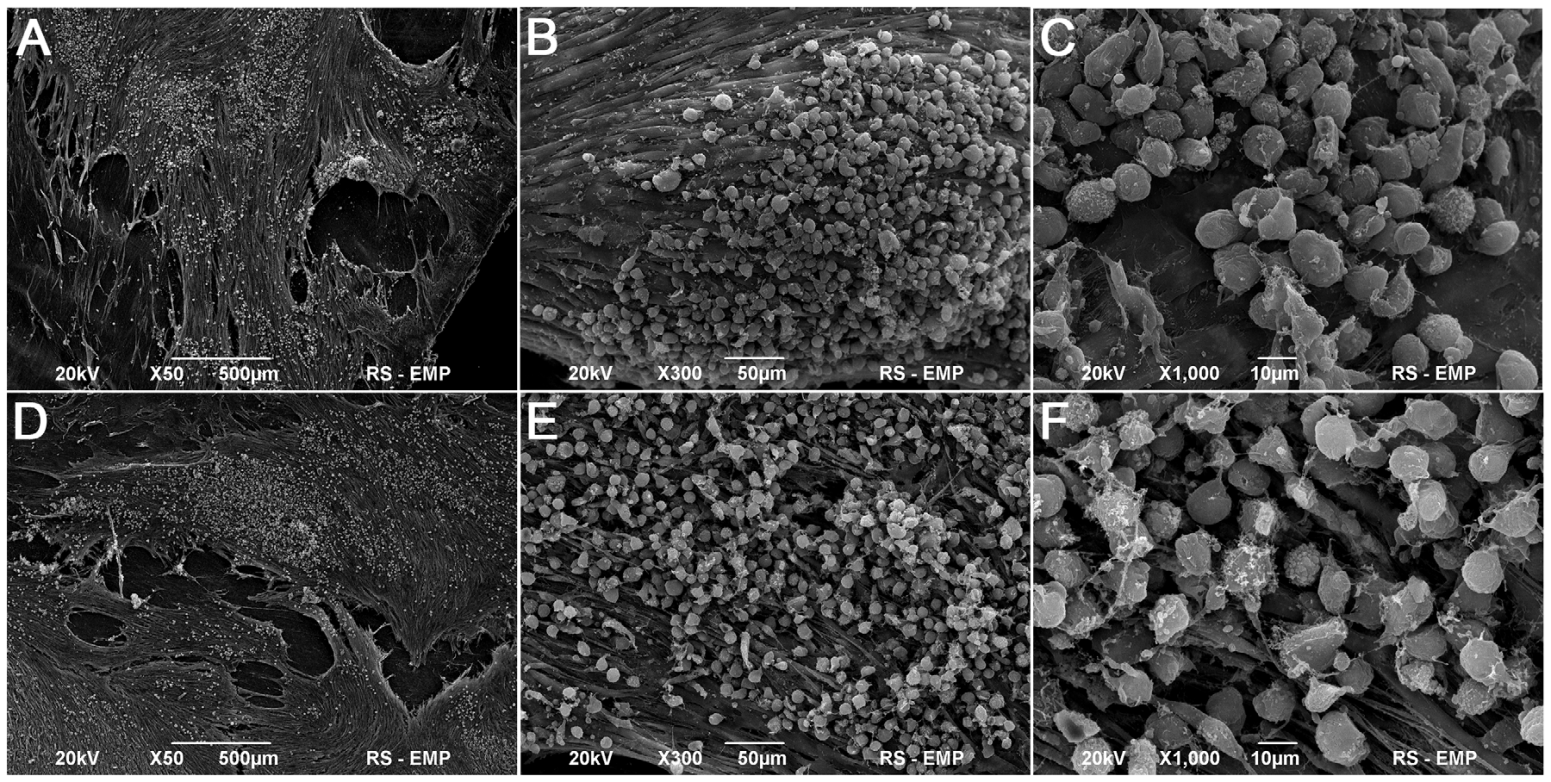
Small intestinal submucosa seeded with adipose derived stem cells (ADSCs), 7^th^ (A,B,C) and 14^th^ day of culture (D,E,F), Scanning Electron Microscopy, bar 500 µm, 50 µm, 10 µm.

ADSCs evenly covered the surfaces of both SIS and PLC membranes (Fig. 6, 7). After 7 days of culturing, the morphology of ADSCs cultured on SIS was normal but only single cells had a flattened shape and elongated cellular processes (Fig. 7A–C). The number of ADSCs adhering to the SIS surface increased after 14 days of culture (Fig. 7D–E). In contrast, a significant number of ADSCs cultured on PLC membrane attached to the biomaterial surface after only 7 days of culture (Fig. 6A–C). The ADSCs had the normal morphology of living cells. The cell divisions were observed. After 14 days of culture, the cells formed a dense layer adhering well to the PLC membrane (Fig. 6D–E).

### Analysis of Urinary Bladder Augmentation

Twelve from sixteen rats survived the 3 months observation time. The highest mortality rate (n = 50%) was observed in PLC groups. Surprisingly, deaths were observed between 2^nd^ and 3^rd^ months of observation. The cause of death was urinary bladder perforation, which occurred within the implanted biomaterial. We found that in many cases the biomaterial implant had twisted, formed a coil and detached from the augmented urinary bladder.

**Figure 8:**
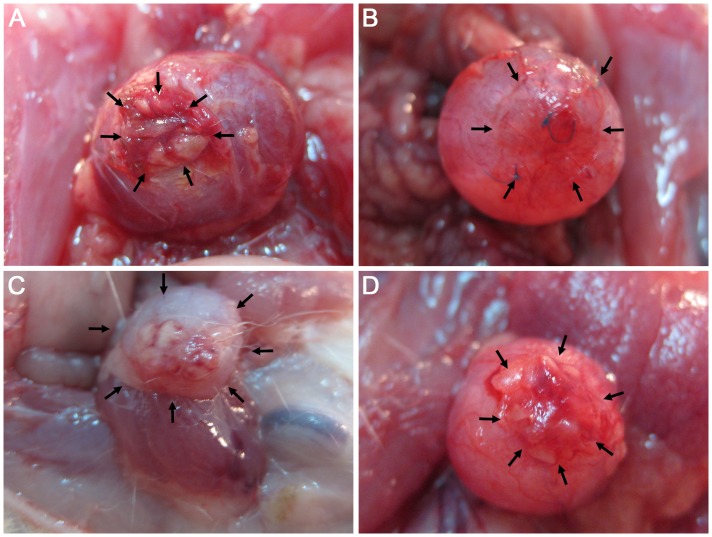
Macroscopic analysis of reconstructed bladders. Bladder augmented with adipose derived stem cells (ADSCs) seeded poly (lactydo-*co*-caprolactone) (PLC) (A), ADSCs seeded small intestinal submucosa (SIS) (B), unseeded PLC (C) and unseeded SIS (D). The arrows point out the reconstructed area.

**Figure 9:**
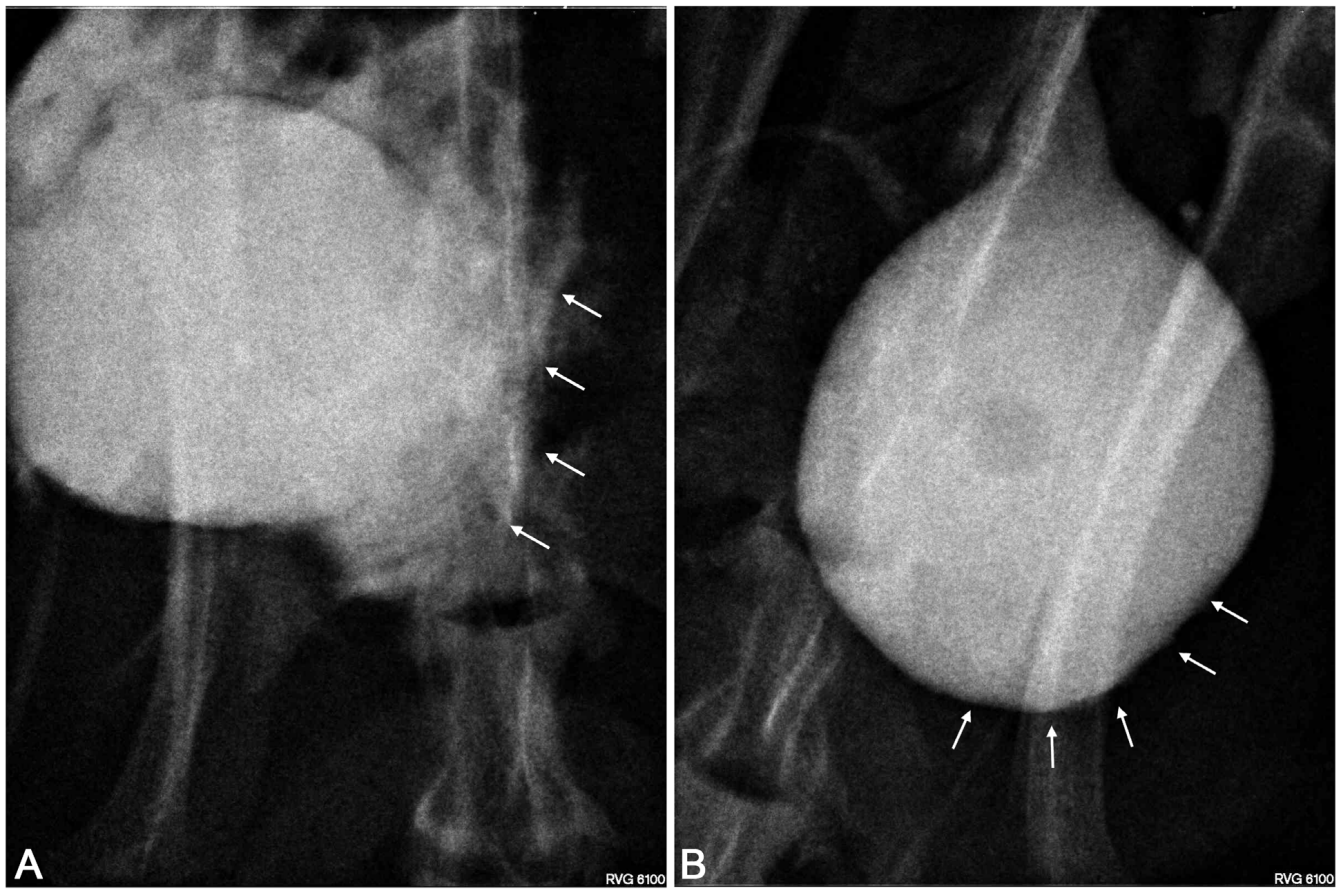
Cystography. Urinary bladders augmented with poly (lactydo-*co*-caprolactone) membrane (A) and small intestinal submucosa seeded with adipose derived stem cells (B). The arrows point out the reconstructed area.

**Figure 10:**
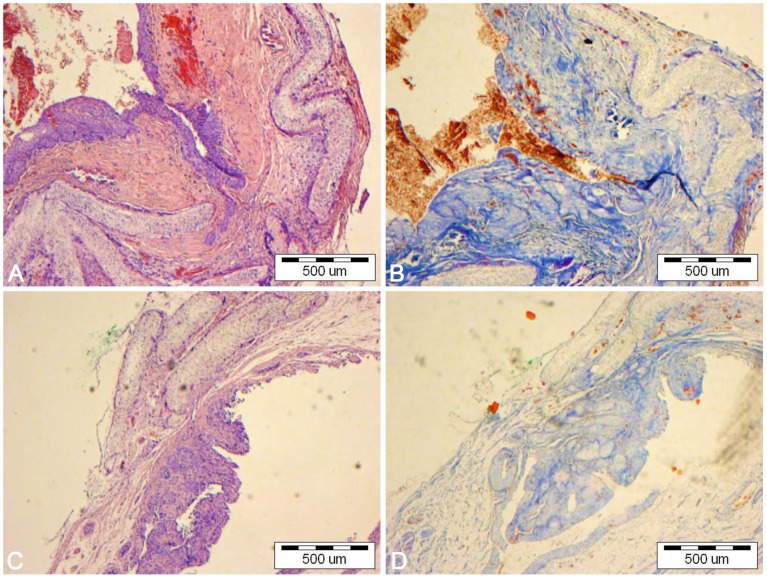
Bladders augmented with poly (lactydo-*co*-caprolactone) membrane seeded (A,B) and unseeded (C,D) with adipose derived stem cells. Light microscope, H&E (A,C) and Trichrome Masson staining (B,D), bar = 500 µm.

Urinary bladder augmentation with the PLC membrane (1^st^ and 3^rd^ groups) led to numerous undesirable events including: perforation, fistula or diverticula formation, and incorporation of the reconstructed wall into the urinary bladder lumen (Fig. 8AC; Fig. 9A). Histological analysis of reconstructed urinary bladders revealed no integration of PLC with bladder tissues (Fig. 10).

**Figure 11:**
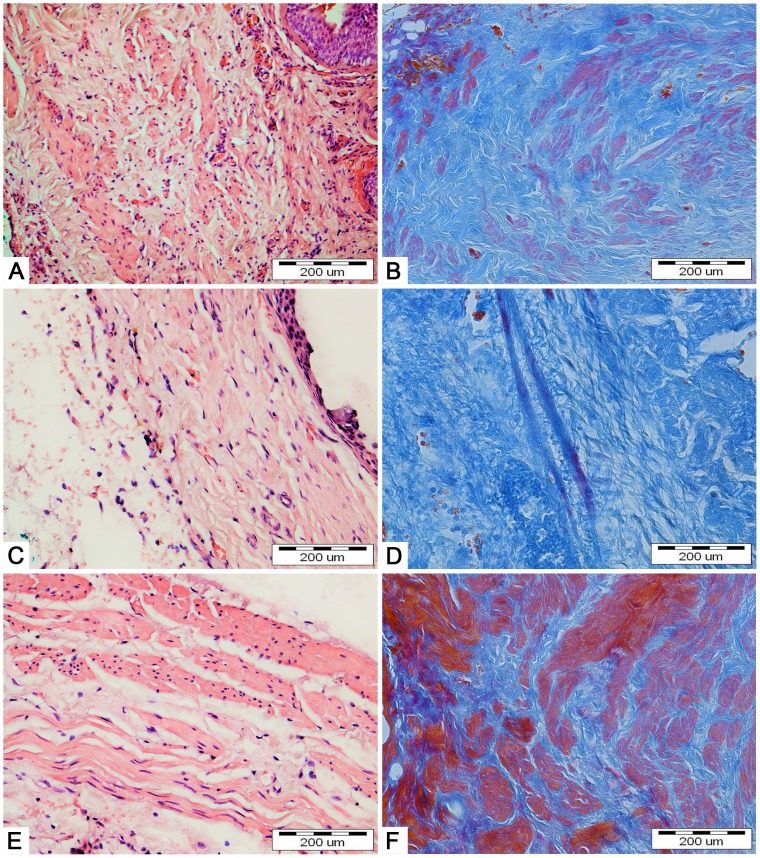
Bladders augmented with small intestinal submucosa seeded (A,B) and unseeded (C,D) with adipose derived stem cells. Native bladders in control group (E,F). Light microscope, H&E (A,C,E) and Trichrome Masson staining (B,D,F), bar = 200 µm.

**Figure 12:**
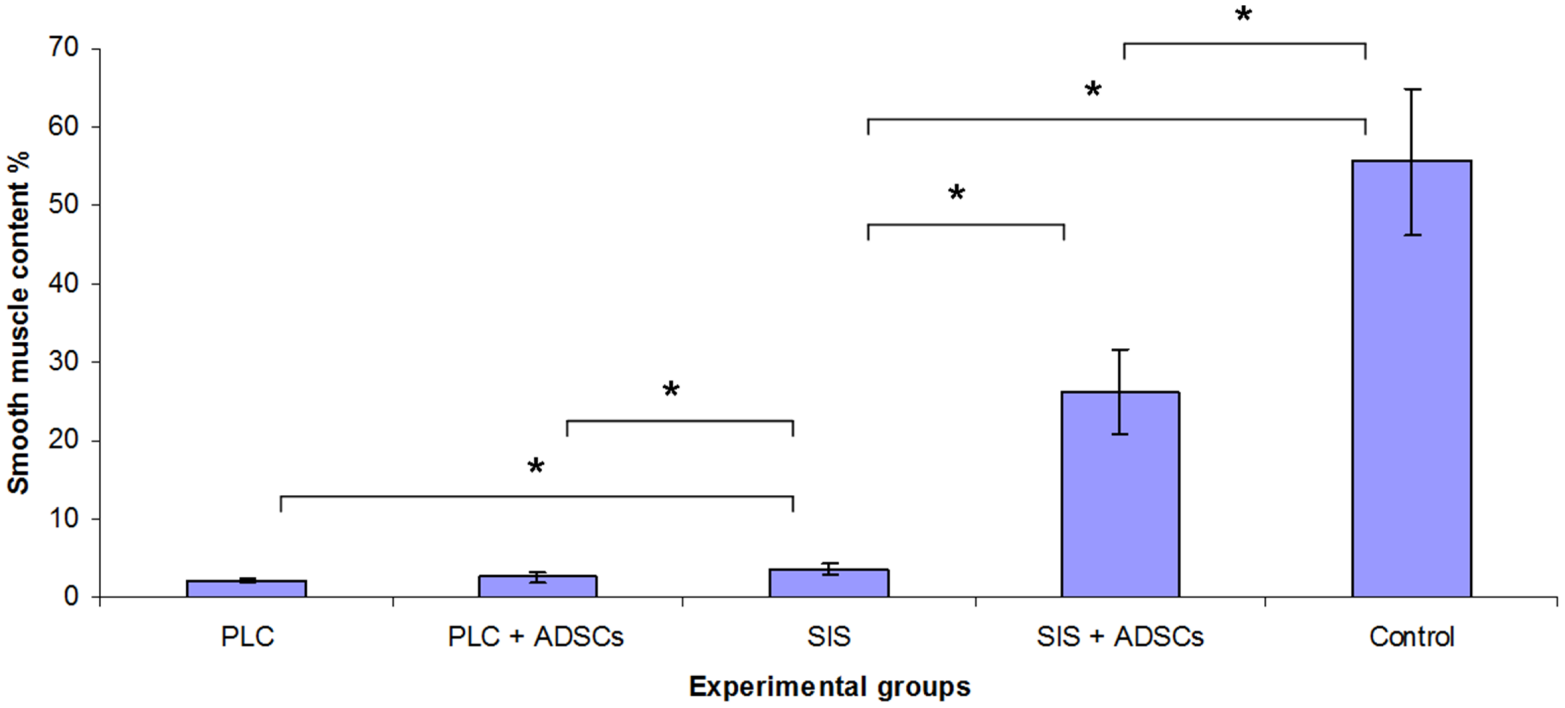
Smooth muscle content in bladders augmented with: poly (lactydo-*co*-caprolactone) (PLC), PLC seeded with adipose derived stem cells (ADSCs), small intestinal submucosa (SIS), SIS seeded with ADSCs and native bladder wall (control), respectively. Values are expressed as mean± standard deviation. The statistical significance is shown as * p<0.05.

Bladders augmented with SIS seeded with ADSCs (2^nd^ group) mimicked native bladders in gross examination (Fig. 8B). Cystography showed proper, regular shape of reconstructed bladders (Fig. 9B). Regeneration of all components of the normal urinary bladder wall was observed. However, smooth muscle fibers were less abundant in the reconstructed wall compared with native bladder wall (26.1±5.4% vs. 55.6±9.3%, p<0.05) (Fig. 11AB, Fig. 11EF, Fig. 12). In the bladders augmented with SIS matrices without cells (4^th^ group), fibrosis and graft contraction occurred (Fig. 8D; Fig. 11CD).

## Discussion

In the present study, the five- layered PLC nanofibrous membrane was produced with the use of a fast-rotating mandrel as a target. Such a type of target ensures a high degree of nanofibers orientation in a specified axis. In order to obtain a membrane of increased strength, a set of 5 orthogonally directed layers of nanofibers was collected. Such a membrane is supposed to have a reinforced structure preventing stretching in all directions. The ultimate tensile strain of PLC membrane was higher compared to rat (2.03 mm/mm, 0.72 MPa) and human (0,69 mm/mm, 0.27 MPa) urinary bladders. Similarly, the elastic modulus of PLC membrane was higher compared to human (0.25 MPa) and rat urinary bladder (0.76 MPa) [27]. These results indicate that the newly synthesized material is elastic and resistant to mechanical treatment. The material was designed to isolate stem cells from the toxic environment of urine. This was achieved because of the high hydrophobicity of the material (both comonomers: L-lactide and ε-caprolactone as well as their copolymer are hydrophobic) and very small air bubbles retained in its structure. However, highly hydrophobic surfaces are not colonized by cells and they cannot sustain cell proliferation. In order to decrease the material's surface hydrophobicity we selected chemical modification of nanofibers [28]–[30]. Other methods are also applied [31], among them are e. g. plasma treatment [32], adhesive protein treatment [33)], silk fibroin modification [34], arginine-glycine-aspartate (RGD) treatment, protein immobilization [35], or surface mineralization with apatite [36]. Our selection was based on the fact that only the surface of the nanofibrous membrane was to be modified while the bulk of the material was left unmodified in order to retain its waterproof properties. A too drastic decrease of hydrophobicity could lead to the loss of isolative properties of the bulk material against the toxic environment of urine, and consequently cause death of stem cells seeded on the nanomaterial surface. The surface of modified electrospun PLC membrane showed improved ADSCs adhesive properties compared with commercially available collagen matrix (SIS). ADSCs adhered to the electrospun PLC membrane and formed a dense layer. Just as in the case of SIS, the PLC membrane pore size was not large enough for cell migration into the scaffold. Such migration was only possible during a subsequent nanomaterial *in vivo* degradation. Further modifications of the porosity of the biomaterial are required. The biomaterial could be degraded *in vitro* before implantation. Another option is synthesis of a biomaterial comprised of layers of different porosity and hydrophobicity. The layer with larger pore sizes and decreased hydrophobicity would allow for directed cell adhesion and migration into the scaffold, while the layer with smaller pore sizes and increased hydrophobicity would isolate the cells from the toxic environment of urine.

We found that the PLC extract did not have a cytotoxic effect on ADSCs in all tested concentrations, as opposed to commercially available SIS extract, which was also confirmed by other authors [37]. In this study, SIS was used as a control because it is the reference material for urinary bladder tissue engineering. To exclude the variables arising from SIS self- preparation we used the commercially available and clinically applicable porcine SIS (Surgisis, Biodesign, USA).

Our *in vitro* analyses showed that the new PLC membrane has better *in vitro* properties compared to SIS, therefore, the use of new PLC membrane in bladder tissue engineering was rational.

Unfortunately, contrary to our expectations the electrospun PLC membrane did not provide an appropriate environment for urinary bladder wall regeneration. There were many differences in regeneration effects between PLC (1^st^ and 3^rd^) and SIS (2^nd^ and 4^th^) groups. The use of adipose derived stem cells did not affect the results of bladder augmentation with PLC membrane but enhanced the smooth muscle regeneration in bladders reconstructed with SIS. Numerous side effects such as: bladder wall perforation, fistula or diverticula formation, or incorporation of the reconstructed wall into the bladder lumen were observed in both PLC groups (1^st^ and 3^rd^). The PLC membrane did not integrate with surrounding bladder tissues. Three months after surgery, PLC membrane degradation was observed, however no signs of remodeling occurred.

Probably, the numerous complications observed in this study result from the multi-layer construction of the membrane. We speculate that a membrane synthesized from non-oriented nanofibrous structure with only a thin layer of directed nanofibers on both surfaces would avoid complications, however it requires future investigation.

In conclusion, the poly L-lactide-co-caprolactone five-layered membrane is not suitable for urinary bladder wall regeneration.
